# Competition and soil resource environment alter plant–soil feedbacks for native and exotic grasses

**DOI:** 10.1093/aobpla/plu077

**Published:** 2014-11-24

**Authors:** Loralee Larios, Katharine N. Suding

**Affiliations:** 1 Department of Environmental Science, Policy & Management, University of California Berkeley, 137 Mulford Hall, Berkeley, CA 94720-3114, USA; 2 Present address: Division of Biological Sciences, University of Montana, 32 Campus Dr HS104, Missoula, MT 59812, USA; 3 Present address: EBIO, University of Colorado, Ramaley N122, Campus Box 334, Boulder, CO 80309-0334, USA

**Keywords:** *Avena fatua*, California grasslands, competition, exotic species, native species, nitrogen enrichment, plant–soil feedbacks, *Stipa pulchra*.

## Abstract

Feedbacks between plants and soil biota are increasingly identified as key determinants of species abundance patterns within plant communities. However, our understanding of how plant–soil feedbacks (PSFs) may contribute to invasions is limited by our understanding of how feedbacks may shift in the light of other ecological processes. Here we assess how the strength of PSFs may shift as soil microbial communities change along a gradient of soil nitrogen (N) availability and how these dynamics may be further altered by the presence of a competitor. We conducted a greenhouse experiment where we grew native *Stipa pulchra* and exotic *Avena fatua*, alone and in competition, in soils inoculated with conspecific and heterospecific soil microbial communities conditioned in low, ambient and high N environments. *Stipa pulchra* decreased in heterospecific soil and in the presence of a competitor, while the performance of the exotic *A. fatua* shifted with soil microbial communities from altered N environments. Moreover, competition and soil microbial communities from the high N environment eliminated the positive PSFs of *Stipa*. Our results highlight the importance of examining how individual PSFs may interact in a broader community context and contribute to the establishment, spread and dominance of invaders.

## Abstract (precis)

Our understanding of how feedbacks between plants and soil microbial communities may contribute to plant invasions and exotic dominance is limited by our understanding of how feedbacks may shift in the light of other ecological processes. In a greenhouse experiment, we found that the strength of plant–soil feedbacks shifted for both a native and exotic as soil microbial communities changed along a gradient of soil nitrogen (N) availability. Moreover, competition from an exotic grass minimized the beneficial feedback between the native grass and its soil microbial community when the soil community was from a high N environment.

## Introduction

Increasingly, feedbacks between plants and soil biota are being identified as key determinants of the abundance and composition of plant communities (Wardle *et al.* 2004; van der Putten *et al.* 2013). Negative feedbacks, where plant species are less productive in their ‘home’ soil biota, are thought to be important in the maintenance of plant diversity (Reynolds *et al.* 2003; Vogelsang *et al.* 2006) and promote species coexistence at small scales. Positive feedbacks, where species are more productive in ‘home’ soil biota, can contribute to species dominance and patch dynamics on a landscape scale (Chase and Leibold 2003; Shurin 2007). Introduced species seem to be exceptions to the rule, as soil biota is often found to have little impact on invasion success (Callaway *et al.* 2004; Inderjit and van der Putten 2010; Suding *et al.* 2013). However within the introduced range, the positive effects of ‘home’ soil biota may contribute to exotic dominance (Grman and Suding 2010). Translating demonstrated plant–soil feedbacks (PSFs) to abundance patterns has had varied results (Klironomos 2002; Yelenik and Levine 2011), as these effects are often considered in isolation from other ecological processes. Environmental factors can affect the dependency of plants on soil biota (Johnson *et al.* 2003) and the composition of the soil communities (Zeglin *et al.* 2007). However, the relative strength of these feedbacks may be small compared with interactions such as plant competition (Shannon *et al.* 2012). Addressing this context dependency of PSFs is key to our understanding of the role of PSFs in plant invasions and exotic dominance.

Soil nitrogen (N) enrichment, via fertilization, atmospheric deposition or other anthropogenic inputs, can alter soil microbial communities (Bissett *et al.* 2013) and facilitate plant invasions (Vitousek *et al.* 1997; Brooks 2003). Despite this evidence, our understanding of how feedbacks may shift in light of these changes to impact plant performance and subsequent invasion dynamics is limited. Under elevated soil N, microbial composition can shift towards a more bacterial dominated community (Bardgett *et al.* 1999; Allison 2002; Bradley *et al.* 2006; Zeglin *et al.* 2007) and can experience a loss of arbuscular mycorrhizal fungal (AMF) species within soil microbial communities (Egerton-Warburton *et al.* 2007; Liu *et al.* 2012). However, the net effect of these soil microbial community shifts on PSFs and invasions is unclear. In addition to these changes in soil microbial communities, host plant identity, which plays a significant role in dictating soil microbial community composition and feedback strength (Bardgett and Cook 1998; Hausmann and Hawkes 2009), can also shift in tandem with resources. For example, native and exotic species loss has been observed with increasing resource availability across multiple grassland systems, but resident natives had a greater likelihood of loss than exotics (Suding *et al.* 2005). Synergistic interactions between shifts in soil microbial communities due to altered resources and shifts in exotic abundance may result in enhanced PSFs that benefit the exotic vs. the native, contributing to invasion; yet these interactive effects are seldom studied.

Plant–soil feedbacks are often assessed at the individual plant level in isolation of other ecological processes such as plant–plant interactions, although they can jointly operate in regulating community diversity and abundance (Hodge and Fitter 2013). Plants can actively secrete compounds within their rhizosphere to promote the acquisition of resources (Hartmann *et al.* 2009), but the presence of the competitor can cause resources to be more limiting and potentially alter the magnitude of PSFs, either intensifying the PSF (Van der Putten and Peters 1997) or eliminating them (Casper and Castelli 2007). Scaling up individual plant responses to soil communities to the community level requires an understanding of how competitive hierarchies may interact with existing PSFs; however, only a handful of studies have investigated both (Van der Putten and Peters 1997; Casper and Castelli 2007; Hol *et al.* 2013) and rarely in the context of invasion (Yelenik and Levine 2011; Shannon *et al.* 2012).

Here, we propose that (i) soil microbial communities from differing resource environments and host plants and (ii) the interaction between plant competition and microbial community can influence the magnitude and direction of PSFs. We focus our study on California grasslands, which have experienced a large-scale shift from native perennial grasses mixed with annual forbs to exotic annual grasses over the last century (Jackson 1985), as well as an increase in atmospheric N deposition (Fenn *et al.* 2003). In this system, annual exotic grasses can shift the composition of soil microbial communities (Hawkes *et al.* 2005, 2006) and can alter the community of AMF colonizing roots of native grasses (Hausmann and Hawkes 2009, 2010), reducing the growth of native species (Vogelsang and Bever 2009).

We conducted a greenhouse experiment where we grew a native, *Stipa pulchra,* and exotic, *Avena fatua* (hereafter, *Stipa* and *Avena*, respectively), in soils inoculated with conspecific (‘home’) and heterospecific (‘away’) soil communities. To examine the interactive effects of resource environment and plant species identity on microbial communities, soil inocula were collected from a field experiment where *Avena* and *Stipa* plots had been treated with either carbon or N addition to alter soil resource availability. To examine the interaction between competitive interactions and microbial function on plant species performance, we grew plants individually or with a neighbour. We hypothesized that if positive PSFs contributed to invasion, then *Avena* would grow better in its ‘home’ soil than ‘away’ soil communities (note: we refer to ‘home’ soil as soils conditioned by the exotic in the introduced range vs. in its native range). Conversely, if *Stipa* were to grow better in its ‘home’ soil compared with ‘away’, positive PSFs would prevent invasion. Moreover, we hypothesized that soil communities from different soil resource environments would contribute to invasion if *Avena* were to grow better with soil communities from high N sites. Lastly, we hypothesized that plant–plant interactions would contribute to invasion if the presence of a competitor weakened the benefit that *Stipa* has when grown in its ‘home’ soil communities.

## Methods

### Study species and soil

We focused on two grass species common to southern California grasslands: the native perennial, *S. pulchra*, and the exotic annual, *A. fatua* (nomenclature follows Baldwin *et al.* 2012). Soils for the experiment were collected from Loma Ridge in Irvine, CA within the Irvine Ranch Land Reserve (N: 33.7501, W: −117.71787)—a grassland largely dominated by a mixture of exotic annual grasses and native perennial grasses (Larios *et al.* 2013). Background soil was collected from this site and upon collection the soil was air dried, sieved through a 2-mm sieve to remove rocks and debris and steam sterilized at 120 °C. This soil was then mixed 1 : 1 with sterile coarse sand and used as the sterile background soil to fill 164 mL cone-tainers for the greenhouse experiment described below.

To test how soil communities from varying N environments affected the strength of PSFs on plant performance, we collected soil inocula in March 2010 from a field experiment where native and exotic plants had been grown separately under low, ambient and high soil N (L. Larios and K. N. Suding, unpubl. data). Within the experiment, N was increased at a rate of 6 g N m^−2^ year^−1^, which we applied in the form of slow-release calcium nitrate (Florikan^®^, Sarasota, FL), and was decreased using table sugar at a rate of 421 g C m^−2^ year^−1^. In similar sites, this level of carbon addition decreased N by ∼30 % (Cleland *et al.* 2013). Soil amendments were applied three times over each growing season, beginning in the 2009 growing season (i.e. 2009 growing season is defined as October 2008 to June 2009) until the end of the 2011 growing season. In total, the experiment consisted of 30 plots (5 replicate blocks × 2 neighbourhood types × 3 soil N). Within each of the five experimental blocks, we collected soils from both the native and exotic plots. Within the native plots, soils were collected directly under a *Stipa* individual and for the exotics, under a stand of *Avena*, ensuring that roots were collected with each soil sample. This soil was kept cool (∼4–6 °C) and shipped to the University of California, Berkeley. Within 3 weeks of collection, the soils from each block were bulked to form the soil inocula used in the experiment. Spatial variation can contribute to high variability in microbial communities within a site (Pereira e Silva *et al.* 2012). Our goal was not to assess this spatial variability by testing the effects of the field soil resource additions on soil microbial communities *per se*, but to ask how soil communities from different resource environments impact plant growth and feedbacks. Therefore, we composited the soils from each block to form the soil inocula used in our soil treatments to ensure that we inoculated with the entire microbial taxa found across a resource environment. We additionally included a sterile soil treatment with no inoculum. Therefore, we had a total of seven soil-community treatments: *Stipa*-conditioned, (i) low N, (ii) ambient N, (iii) high N; *Avena*-conditioned, (iv) low N, (v) ambient N, (vi) high N and (vii) sterile control. The inoculum was added to the cone-tainers at a ratio of 30 : 1, sterile background soil (described above) to inoculum (Bever 1994).

### Experimental design

To assess the interaction between soil communities from different resource environments and plant host on plant–soil interactions in the absence of competitive interactions, we planted three individual seeds of each species by themselves into cone-tainers with the soil inoculated with either conspecific or heterospecific soil communities from low N, ambient and high N sites. To examine the effect of competitive interactions on plant–soil interactions, we also planted species mixtures (consisting of one *Stipa* and one *Avena*) with the seven soil-community treatments described above. After initial germination we removed individuals from all cone-tainers so that each cone had a single individual for the no-competition *Stipa* and *Avena* treatments and one individual of each species for the competitive mixtures. We transplanted seedlings into the cones if no seeds germinated. The transplanted seedlings were planted at the same time as the other seeds so that they were comparable in size upon transplant. Thus we had a total of 420 cone-tainers (7 soil-community inocula × 3 species plantings × 10 blocks × 2 replicates within each block). The multiple replicates within a single block were averaged so that only block means were used in subsequent analyses.

The plants were grown at the Oxford Tract Greenhouse at the University of California, Berkeley, and were watered regularly with distilled water, without supplemental lighting or fertilizer. The blocks were rotated every week to minimize any differential effects of lighting and temperature within the greenhouse. Additionally, the cone-tainers were spaced such that there were never two cone-tainers adjacent to each other, to minimize any potential cross-contamination of soil inocula with watering. All above- and below-ground biomass was harvested 10 weeks after initial planting. Transplanted individuals were harvested 10 weeks after transplanting. The biomass was sorted to species for the competition treatment, and all biomass was dried for 48 h at 60 °C.

### Statistical analysis

To evaluate how plant growth varied across the experiment, we analysed total biomass (sum of above- and below-ground biomass) with a three-way ANOVA, specifying block as a random factor, using the Proc Mixed module (SAS Institute, v 9.1).

We calculated the effect of the soil inoculum pairwise between the sterile soil treatment and the other soil inocula within each block with a natural log-response ratio, ‘ln(*B*_i_/*B*_c_)’, where *B* was the total biomass of the plant in either an inoculated soil treatment (‘i’) or sterile soil (‘c’). We assessed the directionality of the response ratio using *t*-tests, where a value >0 indicated a significant positive response and a value <0 indicated a significant negative response. To assess whether the effect of simply adding soil inocula changed with culturing species or soil resource site, we ran a mixed effects model using the Proc Mixed module separately for each species with the inoculum response ratio as the response variable, soil-community sources (plant species, soil resource site) as two fixed factors and block as a random effect.

To assess whether soil communities from varying soil resources affect plant performance, we calculated for each species a natural log-response ratio (i.e. ln(*B*_alteredN_/*B*_ambN_)), separately for the conspecific and heterospecific soil communities. We then analysed this soil resource response ratio in a mixed model with soil-community sources (i.e. species and soil resource environment) as fixed effects and block as a random effect. We assessed directionality where a positive value would indicate that the individual grew better in the altered soil communities, while a negative value would indicate that it grew worse using *t*-tests as described above. A significant effect of soil resource environment for *Avena* would indicate that the changes in soil communities due to resource environment do alter performance, supporting our second hypothesis. A significant effect of the species soil inocula would indicate whether the effect of the soil communities from varied resourced environments varied between conspecific and heterospecific soil inocula.

Plant–soil feedback strength was calculated as ‘ln(*B*_home_/*B*_away_)’, where *B*_home_ is the total biomass of an individual when grown in their conspecific soil communities and *B*_away_ is the total biomass when grown in heterospecific soil communities. Plant–soil feedback strength was calculated within each soil resource soil microbial community and competition treatment (i.e. *Avena* feedback for no-competition and low N would be the comparison of *Avena* biomass when grown alone, between conspecific (home) and heterospecific (away) cultured soils at low N sites). For blocks where individuals of a specific treatment died, we averaged biomass across the other blocks for that species as a substitute. We did this five times for *Stipa* when grown alone. For the competition treatments, we replaced the biomass of both the species nine times. However, we dropped any blocks that had lost replicates for three or more soil inocula treatments, resulting in a loss of one block for the no-competition treatment and three for the competition treatments.

To assess how PSF responses changed with competition or across soil communities from different soil N environments, we ran a mixed effects model with PSF as the response variable and soil N inocula sources, target species identity and competition as fixed factors. Block was included as a random factor and any significant interactions were evaluated with post-hoc Tukey pairwise difference tests. A significant culturing species–target species interaction would indicate that PSFs could facilitate invasion, if *Avena* experienced no feedbacks when grown in ‘away’ soil communities, but would indicate invasion resistance if *Stipa* experienced positive feedbacks when grown in ‘home’ soil communities. A significant competition–species interaction would indicate that PSFs changed in the presence of a competitor, where a negative shift in feedbacks for *Stipa* when grown in competition would support our third hypothesis.

## Results

### *Stipa pulchra* response

**Figure 1.**
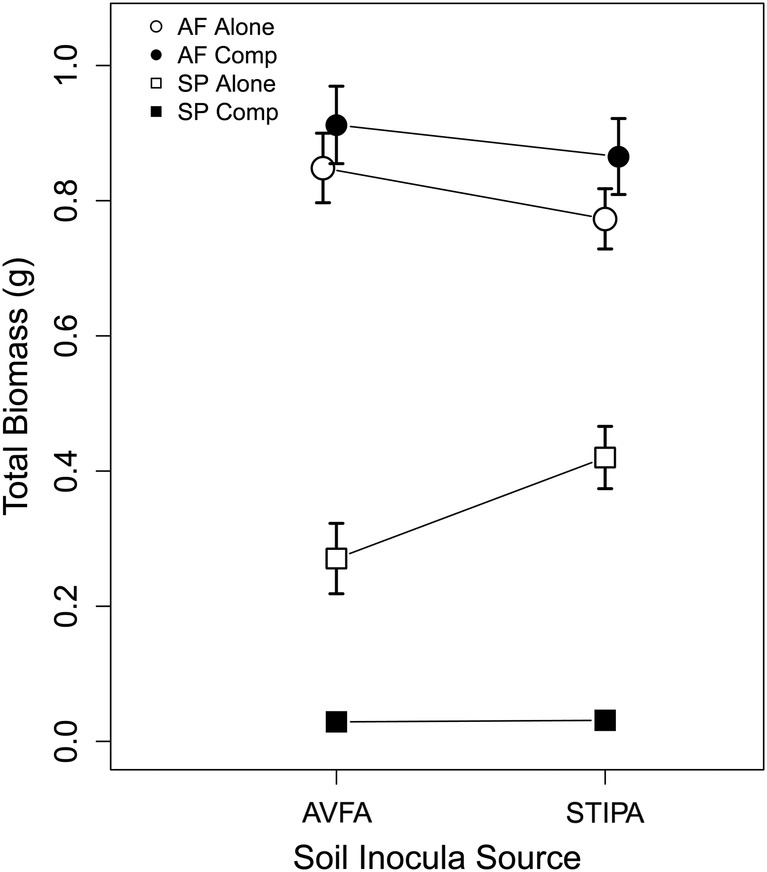
Total (above- and below-ground) biomass for *S. pulchra* (squares) and *A. fatua* (circles) when grown alone (open symbols) or with a competitor (filled symbols) with soil inocula cultured under ambient resources by conspecifics and heterospecifics. Competition decreased *Stipa* biomass, regardless of which soil community *Stipa* was grown. Avena grew similarly in both conspecific (*Stipa*) and heterospecific (*Avena*) soils regardless of the presence of a competitor. Mean ± 1 SE. Error bars for *Stipa* with competitors are hidden by symbols.

**Figure 2.**
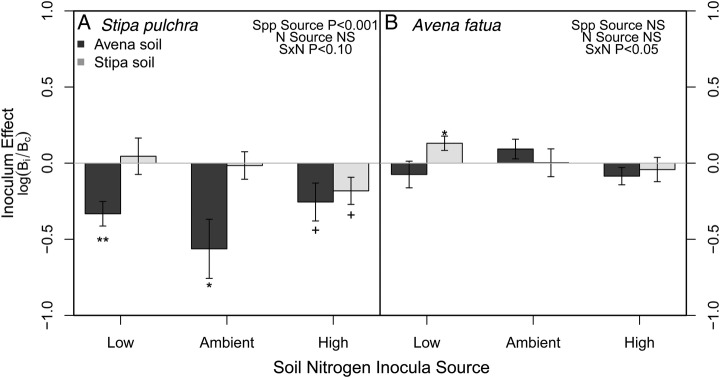
Effect of inoculating soil on plant performance for *S. pulchra* (A) and *A. fatua* (B). *Stipa* experienced negative effects (i.e. grew worse in the inoculated soil treatments compared with sterile) when grown in heterospecific (*Avena*) soil inoculum. Soil inocula affected *Avena* growth only when grown with inoculum from the heterospecific (*Stipa*) grown under low N environments. Mean ± 1 SE. Significantly different from zero: ^+^*P* < 0.07, *P < 0.05, **P < 0.01.

Soil inocula and competitive environment both affected *Stipa* growth. *Stipa* total biomass was affected by soil microbial inoculum from *Avena* and from different soil N environments (culturing species × soil N interaction: *F*_2,76_ = 8.22, *P* < 0.001; [**see Supporting Information**]). Competition decreased *Stipa* biomass by almost 90 % (0.327 vs. 0.036 g, *F*_1,76_ = 595.9, *P* < 0.0001). Additionally, the competitive environment influenced the effect of soil inoculum on *Stipa* (competition × culturing species interaction: *F*_1,76_ = 9.72, *P* < 0.01; Fig. 1, square symbols). Comparisons of growth in sterilized soil indicate that *Avena-*cultured soil communities decreased *Stipa* growth while conspecific-cultured soils had a combination of neutral and negative effects compared with sterilized conditions (culturing species: *F*_1,40_ = 14.18, *P* < 0.0001; soil N: *F*_2,40_ = 0.90, *P* = 0.41; Fig. 2A).

**Figure 3.**
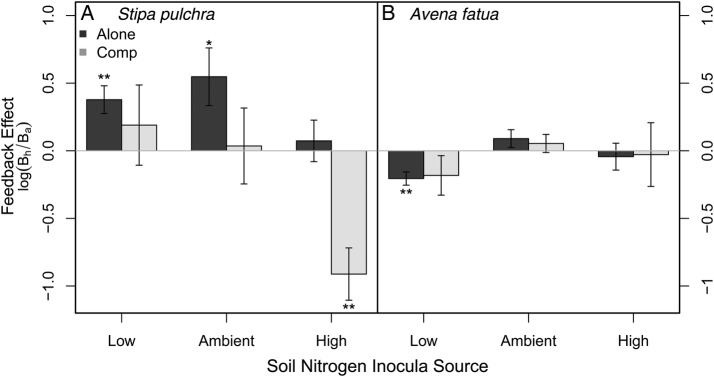
Plant–soil feedbacks for *S. pulchra* (A) and *A. fatua* (B) grown alone or with a competitor, across soils cultured by conspecifics or heterospecifics under varying resources. *Stipa* experienced positive feedbacks (i.e. grew better with its home soil communities) when grown alone in low N and ambient N soil communities, but these feedbacks became negative when grown in high N soil communities. *Avena* grew worse in its conspecific soil compared with heterospecific low N soil communities, resulting in a negative feedback. Means ± 1 SE. Significantly different from zero: *P < 0.05, **P < 0.01.

When grown alone, *Stipa* grew better with conspecific-cultured soil communities compared with heterospecific (better in home vs. away soils), resulting in positive feedbacks when *Stipa* was grown alone (Fig. 3A, dark grey bars). These positive feedbacks diminished when *Stipa* was grown with *Avena* (Spp × Comp, *F*_1,76_ = 7.45, *P* < 0.01; Fig. 3A, light grey bars) and with high N soil communities (soil N × Spp, *F*_2,76_ = 6.24, *P* < 0.01, low and ambient N vs. high N Tukey HSD *P* < 0.01 and *P* < 0.05, respectively), resulting in the development of a strong negative feedback when in competition with *Avena* and in high N soil communities (Fig. 3).

**Figure 4.**
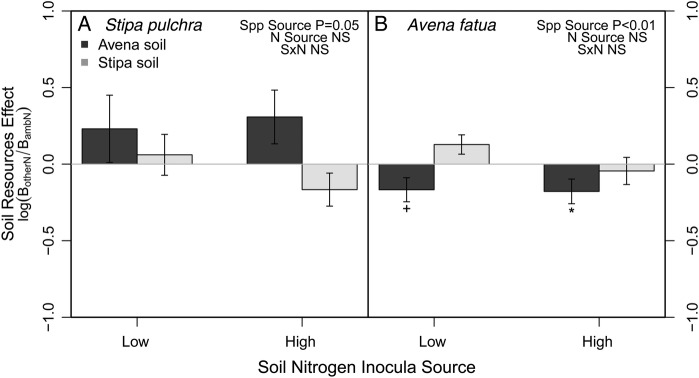
Effect of changes in soil community due to changes in soil nitrogen (N) resources on *S. pulchra* (A) and *A. fatua* (B) growth. *Stipa* grew better in soil communities from ambient N availability compared with low or high N availability when these soils were also cultured by heterospecifics, *Avena.* Conversely, *Avena* grew better in soil communities from ambient N availability when these soils were cultured by conspecifics. Means ± 1 SE. Significantly different from zero: ^+^*P* < 0.07, *P < 0.05.

Soil microbial communities from different N environments did not alter *Stipa* growth; however, *Stipa* grew better with soil communities cultured by the heterospecific, *Avena* (culturing species: *F*_1,24_ = 4.25, *P* = 0.05; soil N: *F*_1,24_ = 0.23, *P* = 0.63; Spp × soil N: *F*_1,24_ = 0.95, *P* = 0.34; Fig. 4).

### *Avena fatua* response

*Avena* exhibited little response to different soil communities (Fig. 3). The only exception to this pattern was a negative feedback at low N, where it grew worse in ‘home’ low N soil communities (soil N × Spp, low vs. ambient N: Tukey HSD, *P* < 0.05). Interactions with *Stipa* did not alter *Avena* growth (*F*_1,76_ = 0.01, *P* = 0.91; Fig. 1, circles) nor change PSFs (Fig. 3). Additionally, *Avena* growth was greater in ‘away’, low N soil communities than under sterile soil conditions (Fig. 2B, culturing species × soil N: *F*_2,40_ = 3.36, *P* < 0.05).

The soil resource environment did not alter the soil community in a way that altered *Avena* biomass. Much like *Stipa*'s response, *Avena* grew better in soils conditioned by heterospecifics compared with conspecifics (culturing species: *F*_1,24_ = 10.22, *P* < 0.01; soil N: *F*_1,24_ = 1.87, *P* = 0.18; Spp × soil N: *F*_1,24_ = 1.45, *P* = 0.23).

## Discussion

Plant–soil feedbacks involve two effects: soil-community effects on plant growth and plant species effects on soil communities (Bever 1994). As such, these feedbacks have most often been studied by isolating these two factors (Kulmatiski *et al.* 2008). However, many other factors can affect the composition of microbial communities (Waldrop *et al.* 2006; Bissett *et al.* 2013), as well as the growth of plant species (Chase and Leibold 2003), leading us to expect that PSFs may be dependent on the broader environmental context (Kardol *et al.* 2013). Indeed, we find that two of these additional factors (soil resource environment effects on soil microbial communities and competitive effects on plant growth) strongly impact the strength and even direction of PSFs.

Plant species effects on microbial communities can strongly regulate species establishment and performance (Bever *et al.* 2010) and the presence or lack of these effects may have strong implications for plant invasions (Inderjit and van der Putten 2010). Here, we observed that the native *Stipa* responded to culturing plant identity, where it grew less in soils conditioned by *Avena,* suggesting that *Avena* is able to culture a distinct soil community that negatively affects the native *Stipa.* On the other hand, we found that *Avena* was not responsive to culturing plant species identity as it grew similarly in soil conditioned by either conspecifics (*Avena-*conditioned) or heterospecifics (*Stipa-*conditioned) compared with sterile soil. While recent reviews have suggested that sterilized and unsterilized comparisons can be biased towards detecting negative responses to soil inocula (Kulmatiski *et al.* 2008; Brinkman *et al.* 2010), the strong response of *Stipa* to soil conditioned by *Avena* suggests that *Avena* may foster soil pathogens at a high enough density to affect *Stipa* growth. Interestingly, we observed an interaction between culturing plant host and soil N environment for both species, but the directionality varied for the native and exotic. *Stipa* grew worse in home soils compared with sterile when the soils came from the high N environment, and *Avena* grew better in heterospecific soils that were cultured at low N compared with sterile soil.

Our results support the idea that resource-induced changes to soil communities can impact PSFs, but the response may be species specific (Manning *et al.* 2008). Across the resource environments, we observed that neither *Stipa* nor *Avena* responded to changes in soil communities conditioned by *Stipa*. However both species responded to shifts in the *Avena*-conditioned soil communities, regardless of whether the conditioning was in low or high N environments, where *Stipa*'s performance improved, while *Avena's* worsened (Fig. 4, dark grey bars). These results support previous findings that *Stipa* is able to foster a more diverse assemblage of soil biota compared with exotic annual grasses (Hausmann and Hawkes 2009), and thus, resource-induced shifts in soil communities may not have a large impact on plant growth. The positive response of *Stipa* to *Avena*-conditioned soil communities in different resource environments has interesting applications for management efforts aimed at native recovery. Soil N reduction activities are traditionally used to alter competitive interactions in favour of the natives (Blumenthal *et al.* 2003), and our results suggest that these soil N reductions may also minimize some of the negative effects on native species' growth that result from the soil conditioning of an exotic species like *Avena*. The small amount of inocula that we used may have resulted in lower densities of harmful pathogens and beneficial symbionts and contributed to the positive/neutral feedbacks that we observed for *Stipa* and *Avena*, respectively (Brinkman *et al.* 2010). However by assessing both the inocula effects and feedback effects, our results suggest that *Stipa*'s positive feedback is likely a result of *Avena* culturing a microbial community that negatively impacts *Stipa.* Additional experiments that explore the spatial variability in the soil community and partition the members of the community to assess the groups driving this pattern are needed to further our understanding of how consistent this response will be across a landscape.

Integrating PSFs into other ecological processes such as competition is key to scaling the impact of PSFs observed at the individual plant level up to the community level (Hodge and Fitter 2013; Kardol *et al.* 2013). Competition had no impact on *Avena* growth, either independently or through a PSF interaction. Independently we observed: (i) when grown alone, *Stipa* grew better in its home soil compared with *Avena*-conditioned soil and (ii) *Stipa* had a strong negative response to competition by *Avena.* However, when we assessed the potential interactive effects of competition and feedbacks, we observed that *Stipa*'s positive feedback was eliminated under competition. While this result is consistent with the competitive hierarchy previously observed between *Avena* and *Stipa* seedlings (Dyer and Rice 1997, 1999), this study does not allow us to decipher whether this result is also due to the strong control that *Avena* species may have on the soil community (Hausmann and Hawkes 2009). The strong effect of *Avena* on *Stipa* performance suggests that restoration efforts should continue to focus on ways to reduce the abundance of exotics in order to promote native species recovery.

Our approach also allowed us to examine how feedbacks may change in the presence of a competitor and soil communities conditioned in different soil N environments. We observed that soils from high N environments eliminated *Stipa*'s positive feedback and interacted strongly with competition such that *Stipa* grew worse in its ‘home’ soil compared with ‘away’ soils. Similarly to the individual effects of soil communities from different resource environments, we observed that *Avena* grew worse in its ‘home’ soil compared with ‘away’ soils. Our results highlight the importance of future studies to explore how PSFs may interact with ongoing environmental change such as atmospheric N deposition to influence the resilience of existing native communities to invasion.

## Conclusions

In conclusion, we found that both plant host and soil resource environment effects on soil communities may alter plant growth and that these impacts can shift in the presence of a competitor. Although the relationships of plant host and soil microbial communities are often assessed in isolation, our ability to understand how they may contribute to observed abundance patterns require us to investigate them in light of other key ecological processes. This more integrated assessment is key to our improved understanding of how plant–soil interactions may contribute to invader establishment, spread and dominance.

## Sources of Funding

This work was supported by the NSF Graduate Research Fellowship Program (DEB 1106400 to L.L.) and NSF (DEB 09-19569 to K.N.S.).

## Contributions by the Authors

Both L.L. and K.N.S. designed the experiment and edited the manuscript. L.L. conducted the data collection and statistical analyses and wrote the first draft of the manuscript.

## Conflicts of Interest Statement

None declared.

## Supporting Information

The following Supporting Information is available in the online version of this article –

**Figure S1.** The average individual total biomass for *Stipa pulchra* (A) and *Avena fatua* (B) as above- and below-ground biomass across soil inocula and competition treatments.

Additional Information
