# Supplementary material for: Competition and soil resource environment alter plant–soil feedbacks for native and exotic grasses
Source: AoB Plants. 2014 Nov 24;7:plu077. doi: 10.1093/aobpla/plu077 (PMC4287689; doi:10.1093/aobpla/plu077)
Supplement: Additional Information [file supp_7_plu077_index.html]

Competition and soil resource environment alter plant-soil feedbacks for a native and exotic grass — Competition and soil resource environment alter plant–soil feedbacks for native and exotic grasses — Additional Information 

# Competition and soil resource environment alter plant–soil feedbacks for native and exotic grasses

## Additional Information

Additional Information

**Files in this Data Supplement:**

- Additional Information - tif file
