# Supplementary figures and images for: Competition and soil resource environment alter plant–soil feedbacks for native and exotic grasses
Source: AoB Plants. 2014 Nov 24;7:plu077. doi: 10.1093/aobpla/plu077 (PMC4287689; doi:10.1093/aobpla/plu077)

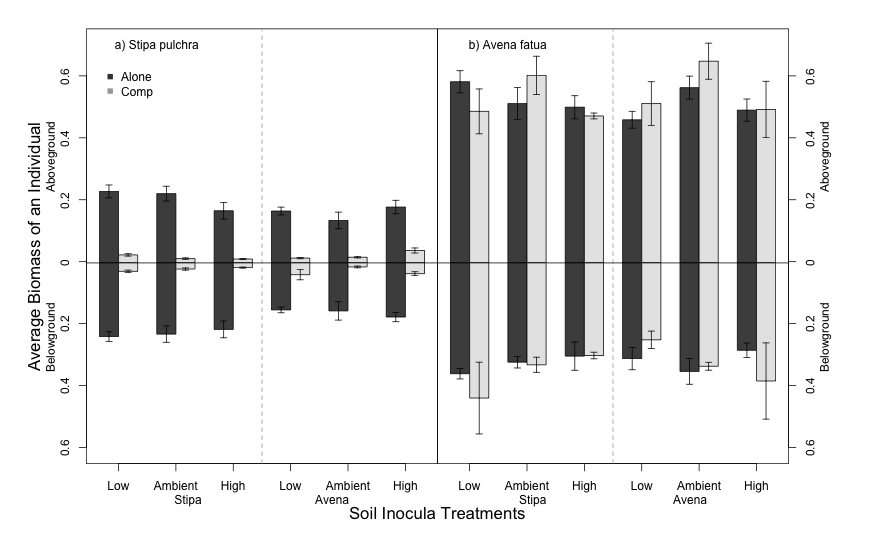

Supplement: Additional Information [file supp_plu077_plu077supp.tif]
